# Chemomechanical regulation of myosin Ic cross-bridges: Deducing the elastic properties of an ensemble from single-molecule mechanisms

**DOI:** 10.1371/journal.pcbi.1005566

**Published:** 2017-05-26

**Authors:** Florian Berger, A. J. Hudspeth

**Affiliations:** 1 Laboratory of Sensory Neuroscience, The Rockefeller University, New York, NY, USA; 2 Howard Hughes Medical Institute, The Rockefeller University, New York, NY, USA; University of California San Diego, UNITED STATES

## Abstract

Myosin Ic is thought to be the principal constituent of the motor that adjusts mechanical responsiveness during adaptation to prolonged stimuli by hair cells, the sensory receptors of the inner ear. In this context myosin molecules operate neither as filaments, as occurs in muscles, nor as single or few molecules, as characterizes intracellular transport. Instead, myosin Ic molecules occur in a complex cluster in which they may exhibit cooperative properties. To better understand the motor’s remarkable function, we introduce a theoretical description of myosin Ic’s chemomechanical cycle based on experimental data from recent single-molecule studies. The cycle consists of distinct chemical states that the myosin molecule stochastically occupies. We explicitly calculate the probabilities of the occupancy of these states and show their dependence on the external force, the availability of actin, and the nucleotide concentrations as required by thermodynamic constraints. This analysis highlights that the strong binding of myosin Ic to actin is dominated by the ADP state for small external forces and by the ATP state for large forces. Our approach shows how specific parameter values of the chemomechanical cycle for myosin Ic result in behaviors distinct from those of other members of the myosin family. Integrating this single-molecule cycle into a simplified ensemble description, we predict that the average number of bound myosin heads is regulated by the external force and nucleotide concentrations. The elastic properties of such an ensemble are determined by the average number of myosin cross-bridges. Changing the binding probabilities and myosin’s stiffness under a constant force results in a mechanical relaxation which is large enough to account for fast adaptation in hair cells.

## Author summary

Myosin molecules are biological nanomachines that transduce chemical energy into mechanical work and thus produce directed motion in living cells. These molecules proceed through cyclic reactions in which they change their conformational states upon the binding and release of nucleotides while attaching to and detaching from filaments. The myosin family consists of many distinct members with diverse functions such as muscle contraction, cargo transport, cell migration, and sensory adaptation. How these functions emerge from the biophysical properties of the individual molecules is an open question. We present an approach that integrates recent findings from single-molecule experiments into a thermodynamically consistent description of myosin Ic and demonstrate how the specific parameter values of the cycle result in a distinct function. The free variables of our description are the chemical input and external force, both of which are experimentally accessible and define the cellular environment in which these proteins function. We use this description to predict the elastic properties of an ensemble of molecules and discuss the implications for myosin Ic’s function in the inner ear as a tension regulator mediating adaptation, a hallmark of biological sensory systems. In this situation myosin molecules cooperate in an intermediate regime, neither as a large ensemble as in muscle nor as a single or a few molecules as in intracellular transport.

## Introduction

The myosin family includes at least 20 structurally and functionally distinct classes [1, 2]. Although they all exhibit a common chemomechanical cycle, myosin molecules have remarkably diverse functions-including intracellular transport, force production in muscles, and cellular migration-as well as important roles in sensory systems [3]. To understand the emergence of these different functions, it is necessary to characterize the biophysical details of the chemomechanical cycle for each myosin class.

Myosin molecules transduce chemical energy into mechanical energy through the hydrolysis of adenosine triphosphate (ATP). The hydrolysis reaction and the subsequent release of inorganic phosphate (P_i_) and adenosine diphosphate (ADP) induce structural changes that result in a power stroke and generate forces. The biochemical reaction rates and the response to external forces determine the specific function of each myosin [3]. On the basis of their biochemical and mechanical properties, myosins have been classified into four groups: (i) fast movers, (ii) slow but efficient force holders, (iii) strain sensors, and (iv) gates [4]. Although single-molecule experiments and structural studies have vastly advanced our understanding of force-producing molecules, we still lack a consistent description that quantitatively relates cellular functions to the molecular details. One prominent case is myosin Ic, which has been identified as a component of the adaptation motor of the inner ear [5].

Hair cells in the inner ear transduce mechanical stimuli resulting from sound waves or accelerations into electrical signals. On the upper surface of each hair cell stands a hair bundle comprising dozens to hundred of actin-filled protrusions called stereocilia. Cadherin-based tip links connect the tip of each stereocilium to the side of the longest adjacent one. When a mechanical force deflects the bundle, the resultant shearing motion raises the tension in the tip links. This tension increases the open probability of transduction channels and allows ions to diffuse into the stereocilia, depolarizing the hair cell.

To retain sensitivity, a hair cell adapts to a prolonged stimulus by changing the tension in the tip links. This adaptation has a fast component lasting a millisecond or less and a slow component of a few tens of milliseconds, the molecular details of which remain uncertain. To explain slow adaptation, it has been proposed that an ensemble of myosin Ic molecules alternately step up or slide down the actin filaments inside the stereocilia to regulate the tension in the tip links. Sliding of myosin is triggered by a locally elevated Ca^2+^ concentration. This picture has been quantitatively supported by experimental studies on hair cells and complemented by mathematical descriptions [6–9]. Fast adaptation describes the rapid reclosure of transduction channels after abrupt stimulation of the hair bundle. This process is poorly understood and several possible explanations at a molecular level are debated [6, 10]. One promising mechanism is the release model, in which a component of the transduction apparatus becomes more flexible and abruptly releases some of the tension in the tip links, allowing the channels to close rapidly [11, 12]. Although myosin Ic has been implicated in both slow and fast adaptation and an ensemble of myosin Ic molecules is a good candidate for the element that releases [10], the precise role of myosin Ic in adaptation has yet to be elucidated.

The rapid response of the transduction channels to a displacement of the hair bundle suggests a direct mechanical activation through the transformation of the deflection into a force by a spring [6, 13]. This mechanism underlies the gating-spring hypothesis that is the prevailing explanation for mechanotransduction by hair cells. The elastic property of the gating spring is the most important parameter in setting the precise relation between the deflection of a hair bundle and the open probability of the ion channels. Despite numerous studies of the molecular components of the hair bundle and their biophysical properties, we remain uncertain of the identity of the gating spring [14–18]. Every molecule that lies in series with the tip link could in principle influence the elastic properties, including the ensemble of myosin Ic molecules. These molecules bind and unbind from actin filaments and thereby change the elasticity dynamically. In order to fully explain mechanotransduction by hair cells, it is important to understand how the dynamics of single myosin Ic molecules determines the elastic properties of an ensemble and how it is regulated.

Over the past few years, the biophysical properties of individual myosin Ic molecules have been characterized in optical traps, biochemical assays, and structural studies [19–24]. Like other myosin isoforms, myosin Ic displays catch-bond behavior, a prolonged attachment to an actin filament in response to increased external force [19, 25]. The force-sensitive step in myosin Ic’s cycle is the isomerization following ATP binding, however, and not ADP release as in other slow myosins [19, 20]. To understand how this behavior relates to the molecule’s physiological function, we introduce a consistent mathematical description of myosin Ic’s cross-bridge cycle.

After the introduction of the basic framework by Huxley and Huxley, cross-bridge models have been widely used to describe the dynamics of myosin motors [2, 26–35]. However, these models often assume irreversible transitions at fixed nucleotide concentrations that determine the input of chemical energy. In a seminal work, T. L. Hill showed how to couple a description of an enzymatic cycle to free-energy transduction in a thermodynamically consistent manner, an approach that has been applied to study muscle myosin [36–39]. We build our cross-bridge cycle for myosin Ic on these concepts and furthermore include the catch-bond behavior.

Our description allows a quantitative analysis of the differences between *in vitro* and *in vivo* conditions, of Ca^2+^ regulation, and of cooperativity between force-producing molecules. Here we introduce a thermodynamically consistent description of myosin Ic based on single-molecule data and focus on the responses to external force, to different nucleotide concentrations, and to the availability of actin. We use this description to predict the elastic properties of an ensemble of myosin molecules and highlight the potential implication for the release model of fast adaptation.

## Results

### Description of myosin Ic’s chemomechanical cycle

**Fig 1:**
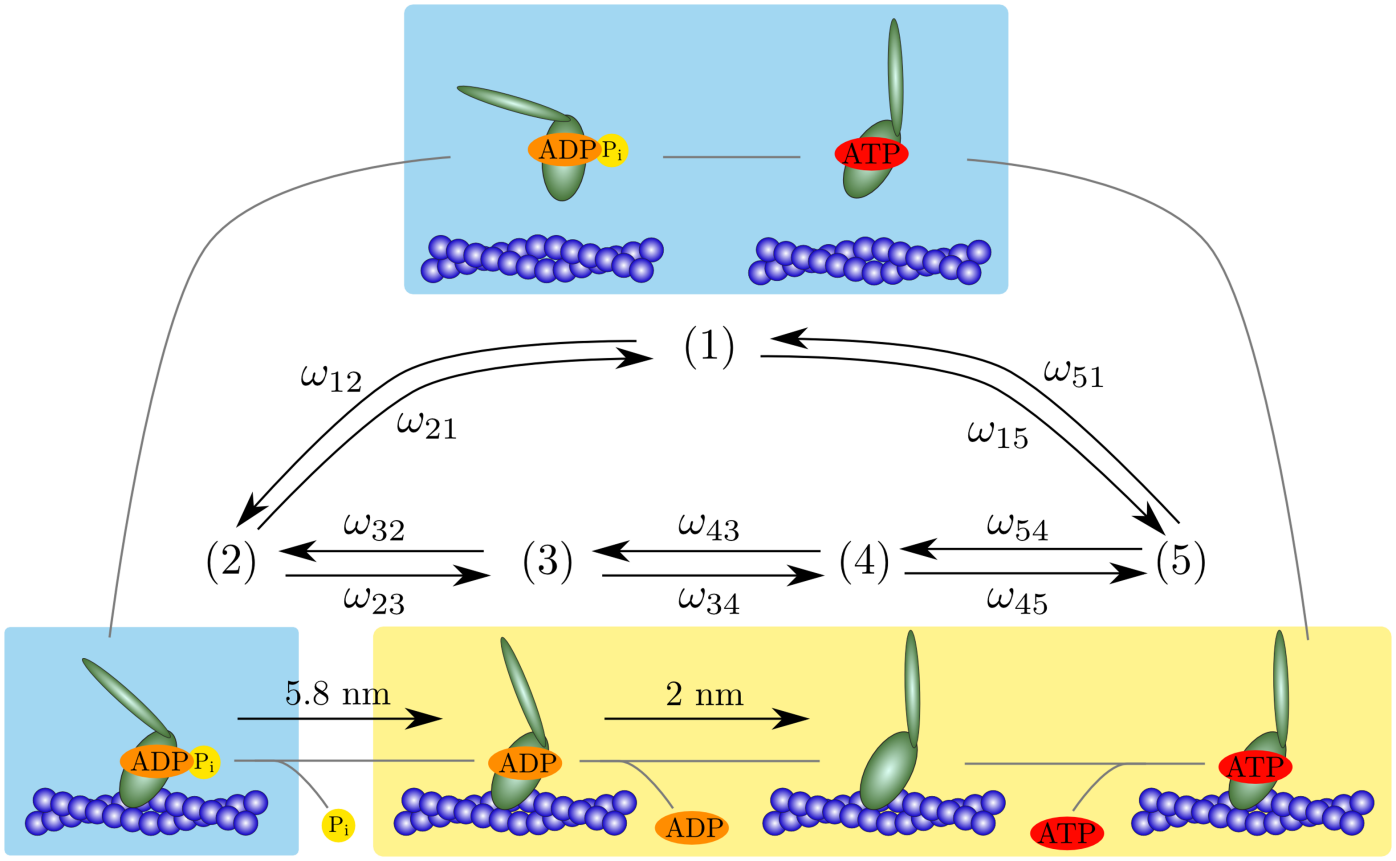
Simplified cross-bridge cycle of myosin Ic. We combine the unbound ADP⋅P_i_ state with the unbound ATP state as a single detached state (1). The four remaining actin-bound states are characterized by the nucleotides bound to the myosin head. The unbound or weakly bound states are shown with a blue background and the strongly bound states with a yellow background. Transitions between the states occur upon the binding and release of nucleotides or upon myosin’s attaching to or detaching from the actin filament. A transition from state (*i*) to state (*j*) is described by the transition rate *ω*_*ij*_.

As a functional description of myosin Ic we introduce a chemomechanical cycle consisting of five states: one state in which myosin is unbound from actin and four actin-bound states. Because we primarily focus on the force-producing states, we consider only a single, effective unbound state that combines the actin-detached ADP⋅P_i_ and ATP states. Each of the actin-bound states is associated with the nucleotide occupancy of the binding pocket of the myosin head (Fig 1). Myosin Ic performs its main, 5.8 nm power stroke upon phosphate release; a smaller power stroke of 2 nm follows ADP release. To account for the work done by these power strokes, we include a force dependence of the associated transition rates. We consider an effectively one-dimensional description in which the force acts along the coordinate of the power stroke: a positive force is oriented in a direction opposite to the power stroke. The nucleotide-binding rates depend linearly on the nucleotide concentrations and the actin-binding rates increase linearly with the actin concentration.

By cycling through the five states, myosin performs work whose magnitude is bounded by the free-energy input associated with the nucleotide concentrations. We base our description on the free-energy transduction of enzymes and thus ensure thermodynamic consistency. To incorporate myosin Ic’s unique force sensitivity, we include a simple force dependence of the rate of unbinding from the filament of myosin in the ATP state. Under high forces, we expect myosin Ic to be trapped in the ATP state. Therefore we consider the ADP state (3), the nucleotide-free state (4), and the ATP state (5) as strongly bound. The remaining states are weakly bound or unbound (Fig 1).

Our description, which captures many of the characteristics of myosin Ic, incorporates as free variables the experimentally controllable quantities external force, nucleotide concentrations, and actin concentration. This approach allows us to obtain analytic expressions for quantities that have been measured in experiments, then to use that information to determine the unknown parameter values of the model. An overview of the parameters is given in Table 1. A mathematical description of the cross-bridge cycle and details of the estimation of parameter values are presented in the Methods section.

**Table 1:** The transition rates and parameter values used in our description. Although the values of most parameters have been measured or can be estimated in the force-free case, the values describing the force dependence have been determined by fitting our model to the experimental data. Note that the transition rate for ATP release is determined by the balance condition of Eq 43 and is not a free parameter of the description. Because the reported values have been measured at room temperature, we use *k*_B_*T* = 4 zJ. Rate constants with a superscribed zero indicate the value in the absence of an external force. The carets denote second-order rate constants with units of s^−1^M^−1^.

Quantity	Symbol	Value	Unit	Source
first power stroke	Δ*x*_1_	5.8	nm	ref. [20]
second power stroke	Δ*x*_2_	2	nm	ref. [20]
ADP release	$\omega_{34}^{0}$	3.9	s^−1^	ref. [20]
eq. constant for ADP release	*K*_ADP_	1.8	*μ*M	ref. [22]
ATP binding	${\hat{\omega }}_{45}^{0}$	0.26	s^−1^ *μ*M^−1^	ref. [20]
P_i_ release	$\omega_{23}^{0}$	1.5	s^−1^	ref. [19, 82]
ADP binding	${\hat{\omega }}_{43}^{0}$	2.17	s^−1^ *μ*M^−1^	Eq 40
actin binding	${\hat{\omega }}_{12}$	0.76	s^−1^ *μ*M^−1^	Eq 35
P_i_ binding	${\hat{\omega }}_{32}^{0}$	4.5 ⋅ 10^−10^	s^−1^ *μ*M^−1^	Eq 38
actin unbinding	*ω*_21_	164	s^−1^	fit
actin unbinding	$\omega_{51}^{0}$	314	s^−1^	fit
actin binding	${\hat{\omega }}_{15}$	0.0015	s^−1^ *μ*M^−1^	fit
force distribution factor	*δ*_1_	0.12	-	fit
force distribution factor	*δ*_2_	0	-	fit
characteristic length	*ξ*	13.45	nm	fit
offset rate	*ω*_off_	0.001	s^−1^	fit
ATP release	$\omega_{54}^{0}$	3.04	s^−1^	Eq 43

### The unbinding rate from the strongly bound states

In a single-molecule experiment using an isometric optical clamp, the lifetime of the myosin Ic-actin bond was measured for different external forces and two sets of nucleotide concentrations [20]. Because a rapid transit into and out of the weakly bound state (2) could not be resolved experimentally, this bound lifetime must be interpreted as the average time *t*_sb_ that myosin Ic spends in the strongly bound states. We determined an analytic expression for the unbinding rate $t_{\text{sb}}^{-1}$ from the strongly bound states (Eq 53) as functions of force and nucleotide concentrations and fit this function simultaneously to two sets of experimental data acquired for distinct nucleotide concentrations. This unbinding rate is independent of the transition rate *ω*_15_ and of the actin concentration. Both quantities determine how often the molecule binds to the filament rather than how long it remains bound. From the average time that myosin Ic resides in the weakly bound states we estimate the binding rate *ω*_15_ for an actin concentration of 100 *μ*M appropriate for the experiments. A detailed explanation for the fitting procedure is given in the Methods section.

**Fig 2:**
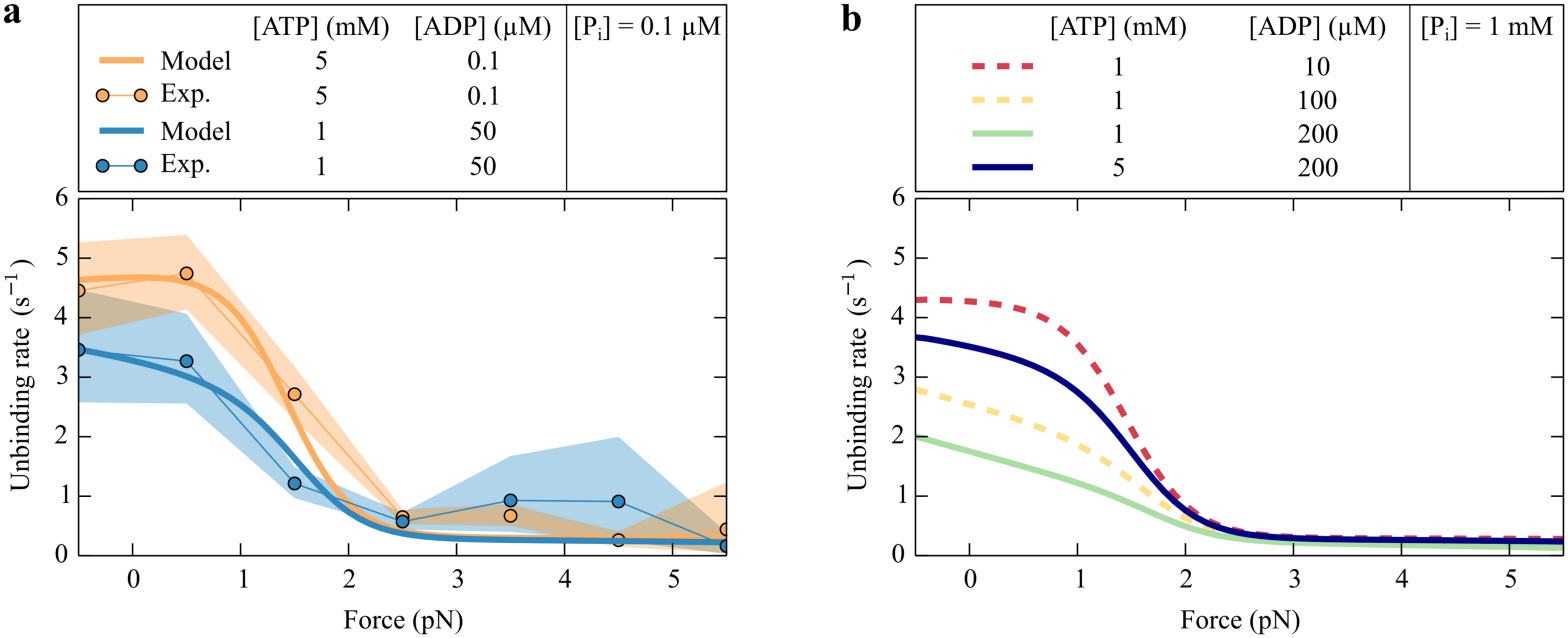
The nucleotide-dependent unbinding rate of a single myosin Ic head from the strongly bound states as a function of force. In (a), we fit the theoretical unbinding rate $t_{\text{sb}}^{-1}$ (Eq 53) (solid lines) to the experimental data (dots) for two different nucleotide concentrations. The orange and blue areas around the experimental data represent the 95% confidence intervals. The parameter values from this fit are summarized in Table 1 and the experimental data have been published [20]. (b) The modeled unbinding rate $t_{\text{sb}}^{-1}$ as a function of force *F* for different nucleotide concentrations. Although an increased P_i_ concentration has no effect compared to (a), raising the ADP concentration reduces the unbinding rate. This reduction can be reversed by increasing the ATP concentration.

Fits of the unbinding rate $t_{\text{sb}}^{-1}$ from the strongly bound states describe the experimental data well, indicating that our description is able to capture the force sensitivity of myosin Ic (Fig 2a). Although none of the transition rates can account individually for the plateau around zero force, their combined effect in the cycle clearly displays such a behavior, which is characteristic of myosin Ic.

The numerical values obtained in this way for the transition rates *ω*_21_ ≃ 164 s^−1^ and $\omega_{51}^{0}≃314\;s^{-1}$ suggest that in the absence of force, state (2) and state (5) are both configurations from which the myosin head rapidly detaches. The force-distribution factors (*δ*) indicate that phosphate release is only weakly dependent on force (*δ*_1_ ≃ 0.12) and ADP release not at all (*δ*_1_ ≃ 0).

The concentrations of nucleotides in cells differ from those in single-molecule experiments. We can use our description to predict the behavior of myosin molecules for different nucleotide concentrations. Although in single-molecule experiments the phosphate concentration usually remains low, the phosphate concentration *in vivo* is on the order of 1 mM [2]. In cells the ATP concentration is also near 1 mM and the ADP concentration is around 10 *μ*M [2]. In the remainder of this study we refer to these numbers as the physiological nucleotide concentrations. The unbinding rate does not significantly change for higher phosphate concentrations (Fig 2a and 2b). The main reason for this robust behavior is the very low rate constant for phosphate binding (Eq 38). Even for a millimolar phosphate concentration the phosphate-binding rate *ω*_32_ is very small compared to the other transition rates in the cycle. In contrast, increasing the ADP concentration decreases the overall binding rate because the molecule spends more time in the ADP state. This effect can be counteracted by an increase in the ATP concentration (Fig 2b).

### Probability distributions of the nucleotide states

**Fig 3:**
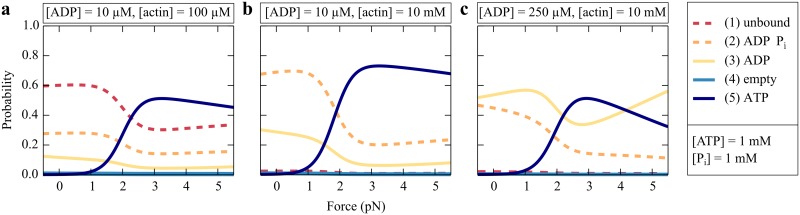
Modeled probability distributions of the states of myosin Ic’s cross-bridge cycle. (a) For physiological nucleotide concentrations and 100 *μ*M actin, myosin Ic is largely unbound from actin under small forces. Considering only the strongly bound states, the ADP state (3) dominates for small forces and the ATP state (5) for forces larger than 1.5 pN. (b) A highly increased actin concentration of 10 mM populates the weakly bound ADP⋅P_i_ state (2) for small forces and myosin is mostly bound to the filament. (c) The occupancy of the ADP state (3) is increased by a raised ADP concentration of 250 mM and dominates for forces smaller than 2 pN and larger than 4 pN.

Using the formulation given in the Methods section with the explicit solutions in Eqs 45–49, we can determine the steady-state probability distribution for the cross-bridge cycle at different nucleotide and actin concentrations (Fig 3). For physiological nucleotide concentrations and 100 *μ*M of actin, myosin is trapped in the ATP state (5) under forces exceeding 2 pN (Fig 3a). Comparing only the strongly bound states, the molecule predominantly occupies the ADP state (3) for forces smaller than 1.5 pN. According to our description, myosin Ic’s cycle through the strongly bound states is limited by ADP release for forces smaller than 1.5 pN and by ATP release for forces larger than 1.5 pN. This result is consistent with experimental findings [19, 20].

In the stereocilium of a hair cell, myosin Ic is thought to extend between the crosslinked actin filaments of the cytoskeleton and the insertional plaque to which the tip link is anchored [5, 6, 40]. To analyze the implications of an environment with a high concentration of actin, we determined the probability distribution for an actin concentration of 10 mM (Fig 3b). Because of the increased binding probability, the unbound state (1) is depopulated. The weakly bound ADP⋅P_i_ state (2) dominates for forces smaller than 2 pN and the ATP state (5) for larger forces. An increased ADP concentration of 250 *μ*M traps the myosin head in the ADP state for forces smaller than 2 pN and larger than 4 pN (Fig 3c). In the intervening regime the ATP state predominates.

### The effective force-velocity relation

In our stochastic description without irreversible transitions, we define myosin’s effective velocity as the average number of forward power strokes minus the average number of reverse power strokes per time. We refer to this definition as an effective velocity to emphasize that this quantity is neither the gliding velocity of an actin filament nor the ensemble velocity of several myosin Ic heads cooperating to produce a continuous movement. Every time the myosin head traverses the states (2) → (3) → (4) it performs a net power stroke of size Δ*x*_1_ + Δ*x*_2_. In contrast, the reverse pathway (4) → (3) → (2) is associated with a reverse power stroke of size −(Δ*x*_1_ + Δ*x*_2_). The effective velocity *v* is accordingly given in terms of the combined local excess fluxes Δ*J*_*ij*_ (Eq 26) as

$$v\equiv \Delta x_{1}\Delta J_{23}+\Delta x_{2}\Delta J_{34}.$$ (1)

**Fig 4:**
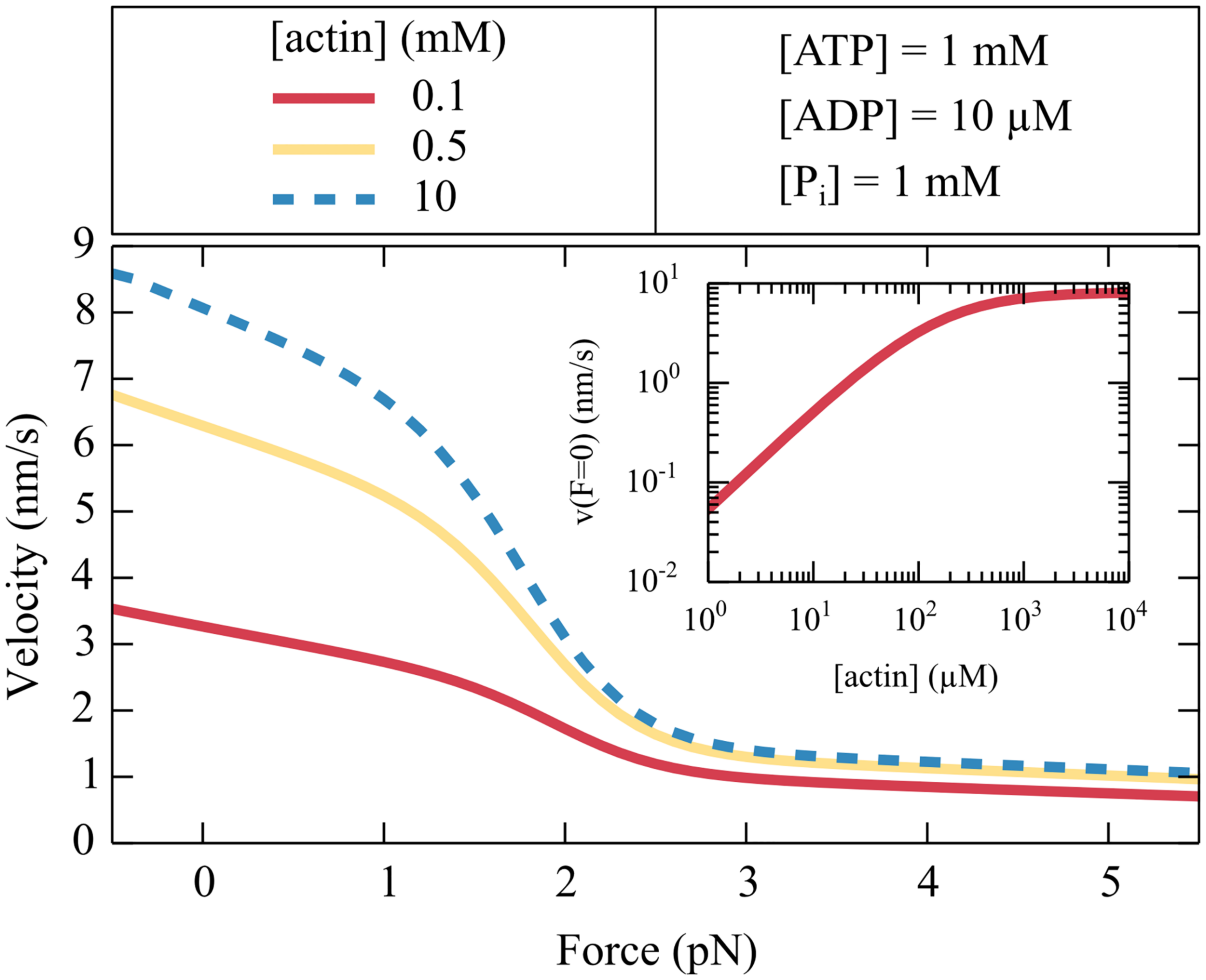
Effective force-velocity relation. The effective velocity *v* depends on the force *F* and on the actin concentrations. A large actin availability enhances the binding of the myosin head and therefore decreases the cycling time, which in turn increases the velocity. The inset shows the effective velocity in the absence of force as a function of the actin concentration.

An increasing actin concentration enhances the binding of myosin and therefore decreases its cycling time, which leads to a higher effective velocity (Fig 4). The velocity saturates for an actin concentration above 1 mM. For large forces the effective velocity decreases until it becomes negative for forces larger than the stall force. According to our thermodynamic description the stall force

$$F_{s}=\frac{k_{B}T}{\Delta x_{1}+\Delta x_{2}}\ln\left(\frac{\left[\text{ATP}\right]K_{\text{eq}}}{\left[\text{ADP}\right]\left[P_{i}\right]}\right)$$ (2)

arises directly from Δ*μ* = *E*_me_, the equality of the Gibbs free energy for the hydrolysis reaction and the mechanical output. This relation reflects an implicit assumption that all of the chemical energy can be converted into mechanical energy. To account for mechanical inefficiency, the description could be extended with a loss parameter. Because we restrict our analysis to forces smaller than 6 pN, for which power strokes have been observed experimentally, we ignore the precise behavior for larger forces and consider the stall force for myosin Ic as an unknown quantity.

### The fraction of time that myosin is bound

A widely accepted definition of the duty ratio is the fraction of the total duration of an ATPase cycle that myosin spends in the strongly bound states [3, 41–43]. Ignoring the weakly bound, actin-attached states or combining them into other states, the duty ratio is often defined as the fraction of the total cycle time during which myosin is attached to an actin filament [2, 44–46]. Because the initiation of myosin Ic’s power stroke is limited by phosphate release, myosin Ic can bind to actin in the ADP⋅P_i_ state but detach without proceeding through the cycle if it detaches prior to P_i_ release. Such an event contributes to the attachment to the filament but not to the time that the molecule spends in the strongly bound states. The time that the molecule spends in the strongly bound states therefore differs from that spent attached to the filament. The probability *P*_sb_ of occupying the strongly bound states accordingly differs from the probability *P*_on_ of being attached to actin. Our complete cycle description allows us to explicitly calculate both probabilities and to compare them. We determine *P*_sb_ in terms of the fraction of the cycle that the molecule spends in the strongly bound states as

$$P_{\text{sb}}\equiv \frac{t_{\text{sb}}}{t_{\text{sb}}+t_{\text{wb}}}=\sum_{i=3}^{5}P_{i},$$ (3)

in which *t*_sb_ is the average time spent in the strongly bound states, *t*_wb_ is the average time spent in the weakly bound and detached states, and *P*_*i*_ is the steady-state probability (Eqs 45–49). Similarly, we obtain *P*_on_ from the fraction of the total cycle time during which the myosin molecule is attached to the filament as

$$P_{\text{on}}\equiv \frac{t_{\text{on}}}{t_{\text{on}}+t_{\text{off}}}=\sum_{i=2}^{5}P_{i},$$ (4)

in which *t*_on_ is the average time that myosin is attached to the filament, *t*_off_ the average time that myosin is detached, and *P*_*i*_ is again the steady-state probability (Eqs 45–49). Whereas the former quantity is closely related to the duty ratio, the later quantity is important for estimation of the number of bound molecules in an ensemble.

**Fig 5:**
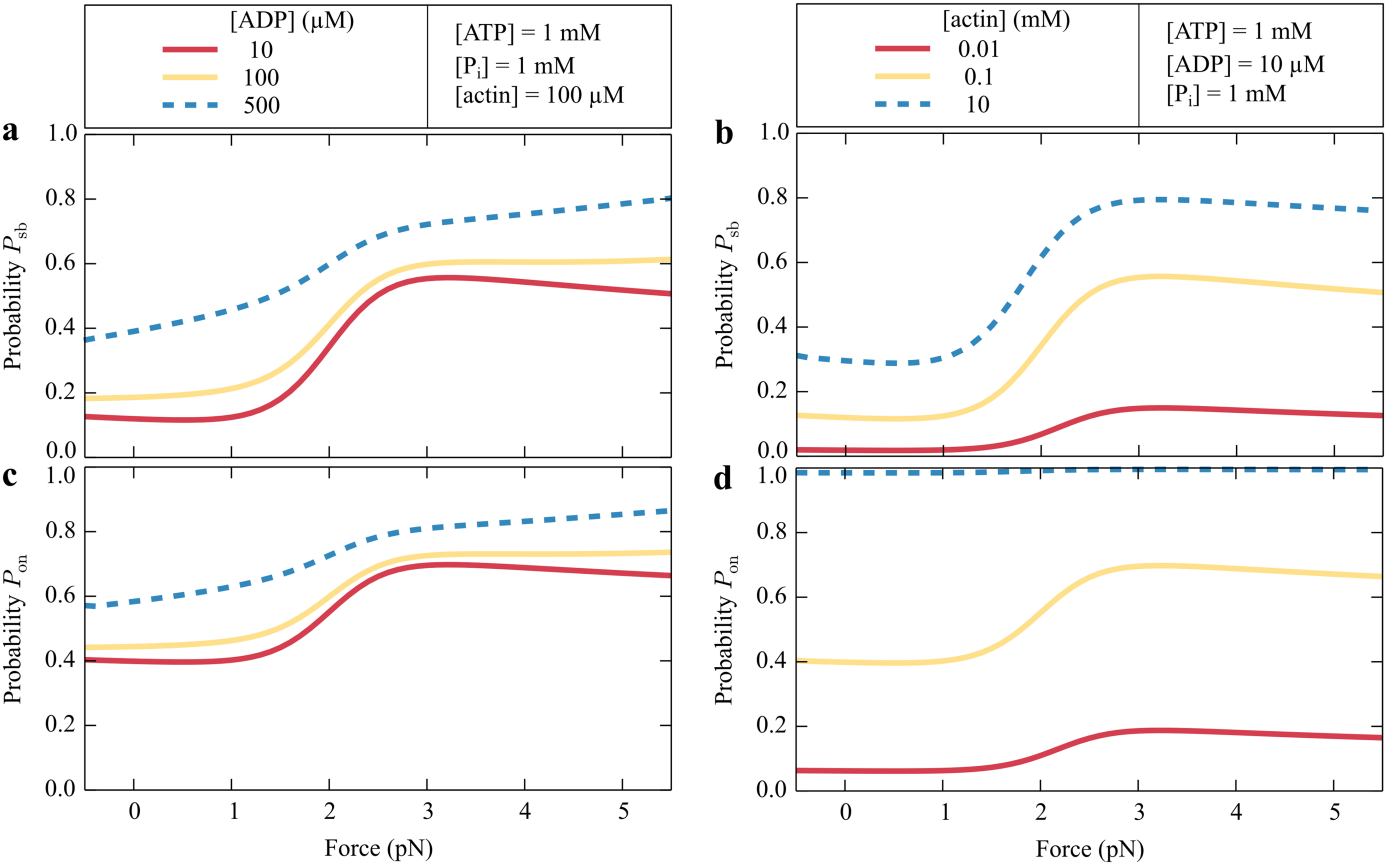
The modeled probability *P*_sb_ of occupying the strongly bound states and the probability *P*_on_ of being bound to actin. (a,c) Changing the nucleotide concentrations or (b,d) the actin concentration is qualitatively similar for probabilities *P*_sb_ and *P*_on_; an increased ADP concentration increases both probabilities. More actin in the vicinity of myosin also enhances both probabilities. However, the probability *P*_sb_ of occupying strongly bound states saturates and is then limited by P_i_ binding, whereas the probability *P*_on_ of being bound to actin reaches unity for high actin concentrations.

The probabilities of being attached to actin and of occupying the strongly bound states depend on the ADP concentration, on the available actin, and on the external force (Fig 5). In general, because of the catch-bond behavior an increasing force enhances the probability of attachment to actin. An elevated ADP concentration likewise traps myosin Ic in the strongly bound ADP state and increases both probabilities (Fig 5a and 5c). An increased accessibility of actin enhances the binding of the myosin head, which results in a high-almost unity-probability of being bound to the filament at high actin concentrations (Fig 5d). In contrast, the probability of occupying the strongly bound states saturates at a high actin concentration, for entering these states is limited by phosphate release (Fig 5b).

### Myosin Ic’s role in the cooperative-release model for fast adaptation

Although in vestibular hair cells myosin Ic activity is required for fast adaptation, the precise molecular details remain unknown [10]. Here we focus on two aspects that might contribute to the mechanism: the cooperative unbinding of an ensemble of myosin heads under force and a qualitative Ca^2+^ dependence that changes the binding probability and the elasticity of individual myosin Ic molecules [23, 24]. In particular, we determine how these properties influence the overall elasticity of an ensemble. The myosin heads contribute to the rigidity of the adaptation motor by crosslinking the insertional plaque to the actin cytoskeleton. We think of each myosin head as a linear spring, arranged in parallel to the others, such that the overall stiffness is given by the sum of the actin-attached myosin heads multiplied by the stiffness of each myosin molecule. Because the binding and unbinding of the heads depend on the force and the nucleotide and actin concentrations, these quantities also influence the overall elastic properties of the ensemble. In general the binding process could be very complicated because of the geometry and possible steric interactions between the heads. Furthermore the helical structure of the actin filaments provides binding sites with an appropriate orientation only about every 37 nm [5]. These constraints change the number of myosin molecules that can potentially interact with actin. In our description, the total number of myosin heads is thus an effective number of molecules that can potentially bind to actin.

**Fig 6:**
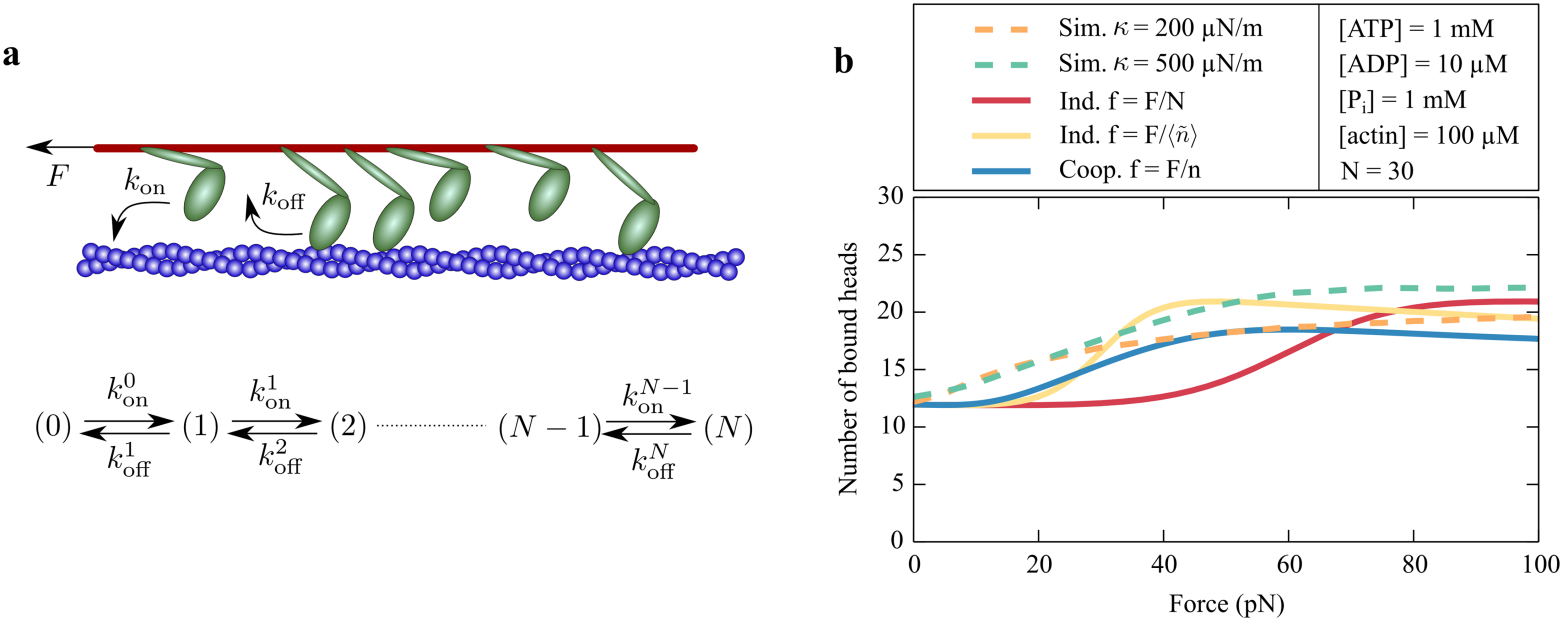
Ensemble of coupled myosin heads. (a) A simplified ensemble model for determining the average number of myosin heads bound to the actin filament. Each of the *N* myosin heads can either bind to the filament with binding rate *k*_on_ or unbind with unbinding rate *k*_off_. The system can be described as a finite Markov chain in which each state is associated with the number of bound myosin heads. The transition rates $k_{\text{on}}^{n}$ and $k_{\text{off}}^{n}$ between these states are effective rates that depend on the number *n* of bound myosin heads. (b) The average number of bound myosin heads for an ensemble of *N* = 30 myosin molecules for a cooperative model (blue, solid line) in which the force per molecule *f* = *F*/*n* is the total external force *F* divided by the number *n* of bound myosin heads. In the two non-cooperative models (red and yellow solid lines) the force per molecule is either constant, *f* = *F*/*N*, or varies according to $f=F/\langle \tilde{n}\rangle$, in which $\langle \tilde{n}\rangle$ is an estimated average number of bound motors. The results of a full Monte Carlo simulation of 30 elastically coupled myosin heads are shown as the broken lines for two different coupling stiffnesses: *κ* = 200 *μ*N/m (orange, broken line) and *κ* = 500 *μ*N/m (green, broken line).

To estimate the average number of bound myosin molecules in an ensemble, we use the attachment and detachment rates determined from our description of the chemomechanical cycle. We assume that each myosin head can bind to the filament with a binding rate *k*_on_ and unbind with an unbinding rate *k*_off_. Both rates stem directly from our description, *k*_on_ = *ω*_12_ + *ω*_15_ and *k*_off_ from Eq 58. Because of the stochastic binding and unbinding, the number *n* of bound molecules fluctuates. To describe the system as a Markov chain, we introduce a state space (Fig 6a) associated with the number of bound myosin heads [47]. The effective transition rates between these states are

$$k_{\text{on}}^{n}\equiv \left(N-n\right)k_{\text{on}},$$ (5)

and

$$k_{\text{off}}^{n}\equiv nk_{\text{off}}.$$ (6)

Here *k*_on_ depends on the actin concentration and *k*_off_ on the nucleotide concentrations and on the force *f* per myosin molecule. We assume that an external force *F* applied to the ensemble is distributed equally among the attached myosin molecules, resulting in the effective force *f* = *F*/*n* per attached head. If one head releases from the filament then the force is redistributed among the remaining bound heads and the force per myosin molecule accordingly increases, which changes the unbinding rate *k*_off_. In general this mechanism leads to cooperative effects because the unbinding rate depends on the number *n* of attached myosin heads. In the case in which the myosin heads act independently, the transition rates of a single head are independent of the number of attached myosin molecules.

We determine the average number of bound myosin molecules from the linear Markov chain as explained in the Methods section,

$$\langle n\rangle =\sum_{n=0}^{N}n{\left[1+\sum_{l=0}^{N-1}\prod_{i=0}^{l}\frac{k_{\text{on}}^{i}}{k_{\text{off}}^{i+1}}\right]}^{-1}\prod_{j=0}^{n-1}\frac{k_{\text{on}}^{j}}{k_{\text{off}}^{j+1}}.$$ (7)

For the cooperative case in which *k*_off_ = *k*_off_(*F*/*n*), we evaluate this equation. In the independent case, in which the unbinding rate *k*_off_ is independent of the number of bound myosin heads, we can simplify this expression to

$$\langle n\rangle =\frac{N}{1+k_{\text{off}}/k_{\text{on}}}=\frac{Nt_{\text{on}}}{t_{\text{on}}+t_{\text{off}}}=NP_{\text{on}}.$$ (8)

Note that *P*_on_ = *P*_on_(*f*) is a function of the force acting on a single myosin head. For the independent case, we estimate this force by *f* = *F*/*N*. However, in this way we underestimate the magnitude of the force per molecule because we expect that *N* > *n*. For a better approximation, we distribute the external force between the mean number of bound motors, *f* = *F*/〈*n*〉, an approach that leads to an implicit equation for 〈*n*〉 that is not easy to solve. For physiological nucleotide concentrations and for 100 *μ*M actin, we notice in Fig 5d that 〈*P*_on_〉 ≈ 0.5. Using this value, we estimate that in a group of 30 molecules about $\langle \tilde{n}\rangle ≃15$ of them are bound on average. We then approximate the average force on a myosin molecule as $f=F/\langle \tilde{n}\rangle$ for the independent case. Note that in the independent case the force per myosin head does not depend on the number of bound heads, in contrast to the cooperative case. The mean number of bound myosin heads is influenced by the cooperative release of the molecules and the three approaches are different for intermediate forces (Fig 6b).

**Fig 7:**
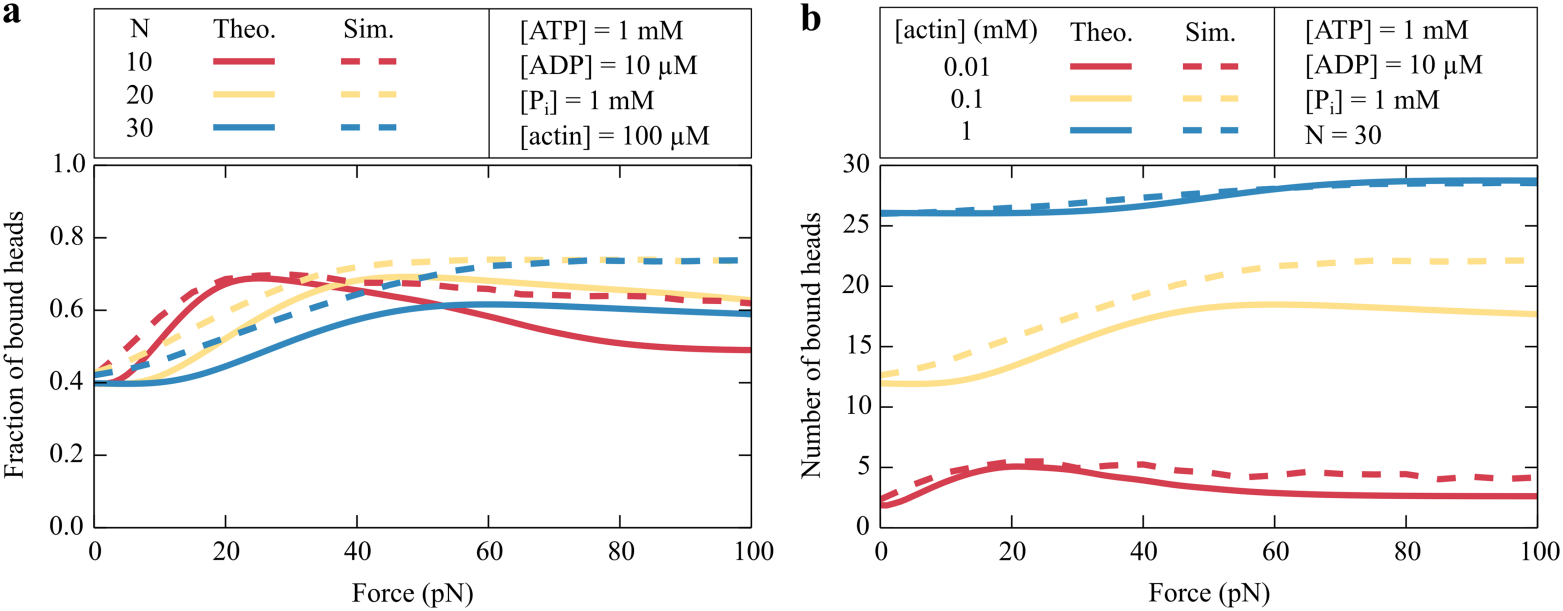
Number of engaged myosin heads. (a) The fraction 〈*n*〉/*N* of bound myosin heads depends on the total number *N* of myosin molecules. A larger fraction of the heads is bound at low forces for a small ensemble than for a large ensemble. (b) In an ensemble of *N* = 30 myosin heads, the average number 〈*n*〉 of bound myosin molecules increases with an increasing actin concentration. In both figures, the analytic results (solid lines) are compared to Monte Carlo simulations (broken lines) with an elastic coupling stiffness of *κ* = 500 *μ*N/m.

We calculate the average number of bound myosin heads as a function of force for different total numbers of myosin molecules (Fig 7a). In small ensembles, the force per head is higher and therefore more heads are bound as a result of the catch-bond behavior. Increasing the concentration of available actin causes more myosin heads to attach to the filament (Fig 7b).

To validate our effective description, we compare our analytic results to Monte Carlo simulations as detailed in the Methods. In these simulations, each myosin head is represented as a spring that is attached to a rigid common structure. At each time step of the simulation the extensions of all springs are calculated by solving Newton’s law of force balance. In this way, we obtain for each myosin head a force that determines the transition rates of the chemomechanical cycle of that molecule. There are important differences from the analytic approach. Whereas in the simulation a myosin head proceeds stochastically through the five-state chemomechanical cycle, the heads only bind and unbind in the analytic description. As a consequence the myosin molecules step stochastically and exert fluctuating forces on each other, which in turn influences their dynamics. In our analytic model, the myosin heads are only indirectly coupled through the number of bound motors and not through an elastic interaction. The simulations show reasonable agreement with the analytic results (Figs 6b and 7). An increased coupling stiffness increases the forces between the myosin heads, which in turn result in a longer attachment because of the catch-bond behavior (Fig 6b). Especially for a high actin concentration, the agreement between the simulations and the analytic description is very good. In the following, we will focus on this particular case and therefore consider only the analytic description.

These results show that the average number of bound myosin heads depends on the external force, the total number of myosin molecules, the actin concentration, and-not shown here-the nucleotide concentrations. We expect that the mechanical properties of a cellular structure including myosin Ic molecules also depend on these quantities.

**Fig 8:**
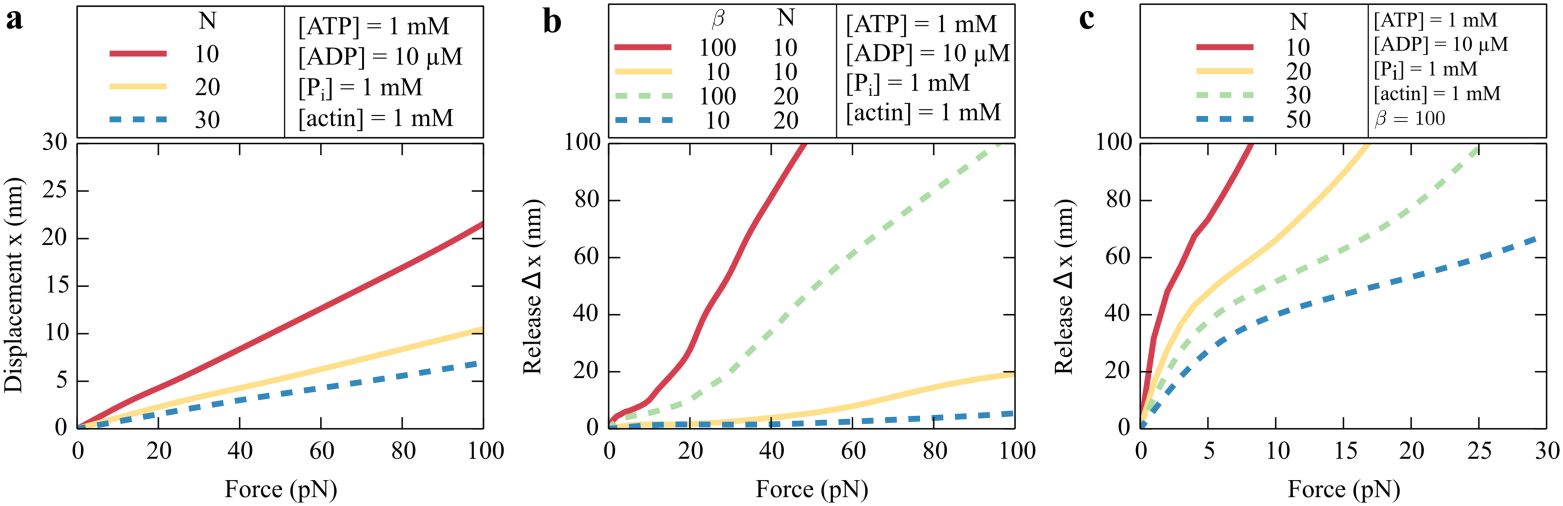
Elastic properties of an ensemble of myosin Ic molecules. (a) The extension *x* of an ensemble of *N* myosin molecules as a function of the applied force. The slope of this effective force-extension relation of an ensemble of myosin Ic molecules increases as the total number of molecules declines. We assume a spring constant for myosin Ic of *κ* = 500 *μ*N/m. Because each head bound to the actin filament contributes to the stiffness, larger ensembles are stiffer. (b) The release Δ*x* after reducing the binding probability by *β*-fold as a function of force for different numbers of myosin heads. For a constant force of 20 pN an ensemble of *N* = 10 heads relaxes about 20 nm after decreasing the binding probability by a 100-fold (red line). (c) The release Δ*x* after reducing the binding probability by 100-fold and the elasticity of individual myosin molecules by 10-fold as a function of force. Increasing the total number *N* of myosin heads weakens the dependence on the force.

To investigate the elastic properties of an ensemble of myosin Ic heads, we determine the force-extension relation

$$F=\langle n\rangle \kappa x,$$ (9)

in which *κ* is the spring constant of a single myosin head. The underlying assumption of this approach is a linear force-extension relation of the individual myosin heads, for which we take the value of *κ* = 500 *μ*N/m [21]. Applying forces below 20 pN to the ensemble leads to an extension smaller than 5 nm (Fig 8a). A reduced total number *N* of myosin molecules increases the extension because the force per myosin head is larger and stretches it farther.

To test whether a mechanical release of myosin Ic molecules is related to fast adaptation, we investigate two qualitative effects of Ca^2+^. First, Ca^2+^ could decrease the binding probabilities of the myosin head to actin [23]. Second, it could change the stiffness of myosin by initiating the dissociation of one or more calmodulin molecules from the light chains, allowing the myosin molecules to attain a more flexible conformation [24].

We next consider the mechanical release owing to Ca^2+^ binding,

$$\Delta x\equiv F\left(\frac{1}{\kappa^{{\text{Ca}}^{2+}}\langle n^{{\text{Ca}}^{2+}}\rangle }-\frac{1}{\kappa \langle n\rangle }\right).$$ (10)

We first study the effect of a reduced binding probability on the mean number of bound myosin molecules and maintain their stiffness before and after Ca^2+^ binding, $\kappa^{{\text{Ca}}^{2+}}=\kappa =500\;\mu \text{N/m}$. We reduce the binding probability by the factor *β* and determine the resulting release for *N* = 10 or *N* = 20 myosin molecules (Fig 8b). A large decrease of the binding probability leads to fewer bound molecules and a larger release. The release for a group of 10 myosin molecules exceeds that for an ensemble of 20 molecules: the force on each individual myosin head is higher and stretches the molecule farther. However, the overall distance for forces smaller than 20 pN is still less than 20 nm. When we add to the 100-fold decrease of the binding probability a tenfold decrease of myosin’s elasticity and determine the resulting release for different total numbers of myosin molecules (Fig 8c), the displacement is of the order of several tens of nanometers and becomes almost insensitive to force for a group of 50 myosins.

## Discussion

An important goal of biology is understanding how the structures and interactions of molecules result in measurable functions of cells and organisms. By combining findings on different spatial scales in a consistent manner, mathematical descriptions help us understand how physiologically relevant function is determined by the interplay of molecular components. We have constructed a quantitative description of myosin Ic’s chemomechanical cycle and studied the resulting properties at both a single-molecule and an ensemble level, which allows us to discuss important implications on the physiological function of hair cells at the whole-cell level.

On the single-molecule level, it is important to understand how different members of the large myosin family display distinct biophysical properties despite a common general structure of the chemomechanical cycle. To describe myosin Ic, we constructed such a cycle and chose as control parameter the nucleotide concentrations and the external force, both of which are experimentally accessible and biologically relevant.

Our simplified, one-cycle description reproduces many of the characteristic features of myosin Ic, especially the force-dependent exit from the strongly bound states. The probabilities of occupying the different states indicate that myosin Ic’s strongly bound states are dominated by the ADP state for forces below 1.5 pN and by the ATP state for larger forces (Fig 3). Although this behavior is in contrast to previous models in which the ADP state is the only force-sensitive state, it is nevertheless consistent with the role of myosin Ic in adaptation [5, 21]. Increasing the ADP concentration traps the myosin heads in the ADP state, bound to actin filaments (Figs 3 and 2b). This effect can be reversed by increasing the ATP concentration (Fig 2b). Such a behavior accords with recordings of transduction currents in hair cells isolated from the bullfrog: changing nucleotide concentrations alters the relative occupancy of the states in the cross-bridge cycle and thus the number of bound myosin molecules, which in turn controls the tension on the mechanically sensitive ion channels. Indeed, in the presence of an ADP analog, adaptation disappears and the tension on the channels increases. Both effects can be reversed by increasing the concentration of ATP [48]. This qualitative agreement constitutes direct evidence that the model, although constructed from single-molecule measurements *in vitro*, captures important aspects of the behavior of living cells.

Our description suggests a low effective velocity for myosin Ic. Although velocities of only tens of nanometers per second have been reported from motility experiments *in vitro* [10, 49–51], larger values have been discussed [5]. In motility assays, multiple myosin molecules work together to create motion. How the velocity measured in motility experiments is related to the effective rate of a cross-bridge cycle and to other biophysical parameters of the molecules is an open question [52–56]. However, our stochastic simulations suggest that 200 elastically coupled myosin Ic molecules, each described by the five-state chemomechanical cycle, display a motility rate of 25 nm⋅s^-1^ which is in good agreement with the experimental values of 16–22 nm⋅s^-1^ [51]. In these experiments the myosin molecules where coupled through a membrane. Greater speeds of 60 nm⋅s^-1^ have been reported in gliding assays, but the data were acquired at a temperature of 37°C [22], whereas the numerical values of the biochemical rates of our model stemmed from experiments conducted at 20°C. We conclude that our description of myosin Ic constrained by single-molecule data accords with the experimental data on a larger scale.

Speeds of tens of nanometers per second are too low to be consistent with rates estimated for the adaptation motor in the inner ear, which has been associated with the function of myosin Ic [5, 10]. Depending on the species, the velocity of the adaptation motor ranges from several hundred to a few thousand nanometers per second [5, 57, 58]. The discrepancy between the velocities *in vivo* and *in vitro* might stem from several factors. It is still unknown to what extent these rates relate to the speed of myosin Ic molecules and to relaxations of other elastic elements. It has been suggested that the recoil of an elastic element located parallel to the myosin heads, the extent spring, contributes to the dynamics [59, 60]. Furthermore the reaction rates of the myosin cycle could be different *in vivo* and *in vitro*. In particular, the complex composition of the cytosol and molecular modifications could lead to differences in the energy barriers between the states [61].

Another possibility is that myosin Ic, which has been shown to constitute the adaptation motor of young mice [10], might be replaced during subsequent development by the closely related paralog myosin Ih, which has been identified as a hair-bundle protein [62]. Myosin Ih’s molecular properties have yet to be characterized and it might operate more swiftly.

In hair cells the deflection by a stimulus is communicated to the transduction channel by an elastic element, the gating spring. Of uncertain origin, this elasticity displays complex behavior with implications for sensory coding [63]. Our model suggests that a cluster of myosin Ic molecules contributes to this elasticity and additionally provide the regulatory function to explain fast adaptation. If we assume that Ca^2+^ reduces the binding probability of myosin by a hundredfold and its stiffness by tenfold, the resultant release on the order of 40 nm accords with measurements from frog hair bundles [10]. For displacements exceeding 400 nm the extent of fast adaptation is independent of the stimulus [10]. We speculate that the insensitivity of the release to the external force for an ensemble of 50 myosin molecules is related to this experimental observation (Fig 8c). Although biochemical studies have suggested that stereocilia contain around 100–200 myosin Ic molecules, the number of actively engaged molecules in an adaptation motor is probably lower [5].

Although myosin Ic’s cycle is slow, binding of Ca^2+^ could rapidly change the relative occupancy of specific states. Under force, most of the myosin heads are trapped in the ATP state. The binding of Ca^2+^ to a myosin Ic molecule triggers the release of calmodulin from the IQ domains and increases the molecule’s flexibility, as recently shown in a structural study [24, 64]. A sudden increase of flexibility would release the myosin head from any force until all elastic elements have relaxed to a new equilibrium state. In the absence of force, our description predicts a transition rate for unbinding from the ATP state as large as $\omega_{51}^{0}≃314\;s^{-1}$. This value is so great that the head would unbind immediately, probably before the forces could be redistributed among the bound myosin molecules. Although the load-free biochemical rates have been reported to be rather insensitive to Ca^2+^, this mechanism might explain a possible Ca^2+^-induced unbinding of myosin molecules from the actin filament under force [23]. Such a fast disengagement of the myosin molecules is necessary for the adaptation motor to slide down the stereociliary actin and thus to relax the tension in the tip links in order to accomplish adaptation to an abrupt stimulus.

Because the transduction channels have been localized at the lower end of the tip links, Ca^2+^ regulation of the adaptation motor is effective only at the next lower insertional plaque [65]. Because the tallest stereocilia lack transduction channels through which Ca^2+^ could enter, the forces between different rows of stereocilia are differently regulated. Inner hair cells consisting of three rows of stereocilia might therefore display less Ca^2+^-regulated slow adaptation [14]. How much of the hair cell’s function is impeded by the reduced regulation is an open question. Hair-bundle models based on detailed descriptions of the relevant molecular mechanisms, such as myosin Ic’s chemomechanical cycle, could provide more insight.

Biochemical experiments and studies of single-molecule motility are ordinarily conducted under chemostatic conditions in which energy sources such as ATP and products such as ADP and Pi are maintained at nearly constant concentrations. In the present study, however, we have endeavored in two ways to model the behavior of myosin Ic under more lifelike conditions. First, we have imposed a thermodynamic constraint that requires the modeled reaction cycles to respect energy balance. And second, we have examined an extensive range of concentrations for the relevant nucleotides and their products. A typical stereocilium, which is about 3 *μ*m in length and 0.2 *μ*m in diameter, has a volume of only 100 aL. Even a substance found at a high concentration in the cytoplasm, such as ATP at 1 mM, can be depleted rapidly in such a small volume. When transduction channels open, for example, the plasma-membrane Ca^2+^ ATPase in a stereocilium confronts a flood of Ca^2+^ that could exhaust the available ATP in only milliseconds! It is thus important to understand the operation of myosin Ic-based motors under realistic and potentially fluctuating conditions.

A final feature of the adaptation motors that remains to be investigated is the noise associated with their activity. By pulling directly on a tip link, each motor influences the opening and closing of the transduction channel or channels at the link’s opposite end. In conjunction with thermal bombardment of the bundle as a whole and stochastic clattering of the transduction channels, the adaptation motors in a hair bundle thus contribute to the mechanical noise that interferes with the detection of faint sounds and weak accelerations [66]. It will be interesting to learn whether the activation mechanism of the myosin molecules in adaptation motors or perhaps their cooperative behavior has been optimized to mitigate this source of noise.

Our study has provided new insights into biological mechanisms. The chemomechanical cycle suggests that the force-dependent unbinding rate is rather robust even under physiological nucleotide concentrations. Although the force-sensitive state is the ATP-bound state, an increased ADP concentration reduces the unbinding rate and the myosin Ic molecules are strongly bound to actin filaments. The elastic properties of an ensemble of myosin Ic molecules can be regulated by an external force and by the actin and nucleotide concentrations. Although the reaction rates of actin-bound myosin Ic are largely insensitive to Ca^2+^ [23], we have shown that in an ensemble of myosin Ic molecules a possible reduction of the binding rate and elasticity could nevertheless account for fast adaptation by hair cells.

## Methods

### Representation of the cross-bridge cycle

The cross-bridge cycle of a myosin molecule consists of distinct states associated with different biochemical compositions and molecular conformations. The transitions between these states involve myosin’s head binding to and unbinding from the actin filament, nucleotide binding and release, and conformational changes. We simplify the cross-bridge cycle and describe myosin’s dynamics with one state (1) in which the head is detached from actin and four states (2)−(5), in which the head is attached (Fig 1). The four actin-bound states correspond to distinct occupancies of the nucleotide-binding pocket: in state (2) ADP and P_i_ are bound, whereas in state (3) only ADP is bound. State (4) is the nucleotide-free state and state (5) refers to the ATP-bound state.

### Transition rates

We represent the cross-bridge cycle as a time-continuous Markov process for which we must specify the transition rates between the states. Although all transition rates could be force- and nucleotide-dependent, it is reasonable to assume that the main effect of the nucleotide concentrations is exerted on the nucleotide-binding rates. Before introducing those transition rates, we discuss the force dependencies of the mechanical transitions.

The transition rates associated with a mechanical power stroke decrease with an increase in the opposing force. Experimental data indicate that myosin Ic performs its power stroke in two steps: the lever arm is remodeled by a distance of Δ*x*_1_ ≃ 5.8 nm upon phosphate release and then by Δ*x*_2_ ≃ 2 nm upon ADP release [20]. Assuming local equilibrium, we associate the ratio of the forward and backward transition rates for the power stroke upon phosphate release with a Boltzmann factor as

$$\frac{\omega_{23}}{\omega_{32}}=\exp\left(-\left(\Delta G_{23}+F\Delta x_{1}\right)/k_{B}T\right).$$ (11)

Here Δ*G*_23_ is the Gibbs free-energy difference between the states, *F*Δ*x*_1_ is the mechanical work performed by the power stroke of distance Δ*x*_1_ against the opposing load force *F*, and *k*_B_ *T* is the Boltzmann constant times the temperature [67]. The equation above relies on an assumption of local equilibrium that does not indicate how the individual transition rates depend on the force. Therefore, we use the following general forms for the individual transition rates:

$$\omega_{23}\equiv \omega_{23}^{0}\exp\left(-\delta_{1}F\Delta x_{1}/k_{B}T\right),$$ (12)

$$\omega_{32}\equiv \omega_{32}^{0}\exp\left(\left(1-\delta_{1}\right)F\Delta x_{1}/k_{B}T\right),$$ (13)

in which we introduce the force-free rate constants $\omega_{23}^{0}$, $\omega_{32}^{0}$ and the force-distribution factor *δ*_1_ ∈ [0, 1]. The restriction on the numerical values for the force-distribution factor is a consequence of the assumption that a force opposing the power stroke diminishes the corresponding transition rate [67, 68]. For an effective description based on a projection of a high-dimensional free-energy landscape on to a single reaction coordinate, the force-distribution factor is not restricted [69]. Using the same argument as for the release of phosphate, the general forms of the forward and backward transition rates associated with the power stroke upon ADP release are

$$\omega_{34}\equiv \omega_{34}^{0}\exp\left(-\delta_{2}F\Delta x_{2}/k_{B}T\right),$$ (14)

$$\omega_{43}\equiv \omega_{43}^{0}\exp\left(\left(1-\delta_{2}\right)F\Delta x_{2}/k_{B}T\right).$$ (15)

To account for the force-dependent behavior of myosin Ic, we must include the force sensitivity of the isomerization following ATP binding [19]. In our simplified state space this sensitivity effectively changes the unbinding rate *ω*_51_ from the ATP state. We therefore introduce a force-dependent factor *g*(*F*) that modifies the unbinding rate

$$\omega_{51}\equiv g\left(F\right)\omega_{51}^{0}.$$ (16)

We require that for zero force *g*(*F* = 0) = 1 and for large force *g*(*F* ≫ 1) = *ω*_off_ saturates and use

$$g\left(F\right)\equiv \frac{2(1-\omega_{\text{off}})}{1+\exp(\xi F/k_{B}T)}+\omega_{\text{off}},$$ (17)

in which *ξ* is a characteristic length scale.

In general the binding interface between the head of a molecular motor and its filament is more complicated than the idealized receptor-ligand bond considered by Bell [70]. The bond interface consists of multiple partial charges that lead to complex unbinding pathways through multiple states in the free-energy landscape [71–74]. To capture the characteristic behavior, we use the force factor of Eq 17 that has been used previously to describe the chemomechanical cycle of kinesin-1 and myosin V [75–79].

In the following we give an intuitive justification of the force factor given in Eq 17. In our description the ATP state (5) comprises several sub-states including the binding and isomerization of ATP. The unbinding rate *ω*_51_, which must be considered as an effective rate for proceeding through all the sub-states, therefore includes the force dependence of the isomerization step. As a first approximation, we consider that isomerization is not associated with a conformational change that would result in a displacement of an applied load. The free-energy between the state (*A*) before isomerization and the state (*B*) after isomerization accordingly does not depend on the applied force. We assume that an applied force increases the free-energy barrier between those two states without changing the difference of the energy between the states. Motivated by Kramers rate theory [80], we use the force dependence $\omega_{AB}=\omega_{AB}^{0}\exp\left(-F\xi /k_{B}T\right)$ for the forward transition rate and the same force dependence for the reverse transition rate $\omega_{BA}=\omega_{BA}^{0}\exp\left(-F\xi /k_{B}T\right)$. We consider the main forward pathway through these sub-states,

$$\to \left(A\right)\underset{\omega_{BA}}{\overset{\omega_{AB}}{⇌}}\left(B\right)\overset{\omega_{B}}{\to },$$ (18)

which implies the effective transition rate

$$\omega_{\text{eff}}=\frac{\omega_{B}}{1+\frac{\omega_{BA}^{0}}{\omega_{AB}^{0}}+\frac{\omega_{B}}{\omega_{AB}^{0}}e^{F\xi /k_{\text{B}}T}}.$$ (19)

This approach leads to a force dependence similar to that in Eq 17. Using the same argument for the reverse pathway, we find that the transition rate *ω*_54_ has a similar force dependence. As described later in Eq 43, the force dependence of the transition rate *ω*_54_ is imposed naturally by thermodynamic consistency.

To capture the dependence on the nucleotide concentrations, we consider the nucleotide-binding steps as first-order reactions that are independent of force, leading to

$$\omega_{32}^{0}\equiv {\hat{\omega }}_{32}^{0}\left[P_{i}\right],$$ (20)

$$\omega_{43}^{0}\equiv {\hat{\omega }}_{43}^{0}\left[\text{ADP}\right],$$ (21)

$$\omega_{45}\equiv {\hat{\omega }}_{45}^{0}\left[\text{ATP}\right].$$ (22)

Note that the units of the rate constants with a caret are M^−1^s^−1^. Such a linear dependence of the transition rates on the reactants is motivated by macroscopic chemical-reaction laws and is widely used to describe chemomechanical cycles [2, 67]. In a similar way, we assume a linear dependence of the actin-binding rates on the actin concentration,

$$\omega_{12}\equiv {\hat{\omega }}_{12}\left[\text{actin}\right],$$ (23)

$$\omega_{15}\equiv {\hat{\omega }}_{15}\left[\text{actin}\right].$$ (24)

The remaining transition rate *ω*_54_ is determined by a balance condition obtained from thermodynamic consistency.

We have specified above the general forms of the transition rates of our theoretical description of myosin Ic. We next introduce the dynamics and the thermodynamic constraints. We consider the stochastic dynamics of the myosin head as a continuous-time Markov process [81]. The probability *P*_*i*_(*t*) of finding myosin in state (*i*) therefore evolves in time *t* according to the master equation

$$\frac{d}{dt}P_{i}\left(t\right)=-\sum_{j}\Delta J_{ij}\left(t\right),$$ (25)

with a local net flux between the states (*i*) and (*j*) given by

$$\Delta J_{ij}\left(t\right)\equiv P_{i}\left(t\right)\omega_{ij}-P_{j}\left(t\right)\omega_{ji}.$$ (26)

A thermodynamically consistent description, which ensures that myosin does not produce more mechanical energy than the chemical energy provided by the nucleotide concentrations, implies a relation between the mechanical energy and the chemical energy. This relation provides a constraint on the transition rates of the cycle that is obtained by incorporating free-energy transduction [37]. We can express the change in the Gibbs free energy of the hydrolysis reaction for a dilute solution by

$$\Delta \mu \equiv k_{B}T\ln\left(\frac{\left[\text{ATP}\right]}{\left[\text{ADP}\right]\left[P_{i}\right]}K_{\text{eq}}\right),$$ (27)

in which *K*_eq_ is the equilibrium constant for the reaction, here with the numerical value *K*_eq_ ≃ 4.9 ⋅ 10^5^ M [2]. Note that at the equilibrium concentration of the nucleotides the change in the free energy Δ*μ* vanishes.

The mechanical energy, which is the work done by the protein against an external force *F*, is given by

$$E_{\text{me}}\equiv \left(\Delta x_{1}+\Delta x_{2}\right)F.$$ (28)

This mechanical energy is produced when the protein passes through a forward cross-bridge cycle, which in our description represents directed transitions through the states (1), (2), (3), (4), (5), and finally (1). In contrast, the backward cycle is associated with a path traversed in the opposite direction. Thermodynamically consistent coupling of the energy conversion by the protein to the hydrolysis reaction then imposes the constraint

$$\frac{\omega_{12}\omega_{23}\omega_{34}\omega_{45}\omega_{51}}{\omega_{21}\omega_{32}\omega_{43}\omega_{54}\omega_{15}}=\exp\left(\left(\Delta \mu -E_{\text{me}}\right)/k_{B}T\right),$$ (29)

which can further be related to the entropy production [37, 77]. This equation has an intuitive interpretation [37]: the left side is the ratio of the average number of complete forward cycles to the average number of complete backward cycles, whereas the right side is the exponential of the difference between the chemical input energy and the mechanical output energy. At equilibrium this difference vanishes and the right side is equal to one, which requires the completion of identical numbers of forward and backward cycles. This constraint ensures that the average net cycling of the protein is thermodynamically consistent with the energy input. In our simple approach each hydrolysis reaction produces a power stroke, meaning that there are no futile cycles and therefore the chemistry is tightly coupled to the mechanics.

### Specification of transition rates and parameter values

We incorporate into our description of myosin Ic as many experimental data as possible. Some transition rates and parameter values have been reported [19, 20]; an overview of these is given in Table 1. The force-dependent lifetime of an actin-myosin bond has been determined with an optical trap [20]. Because of the finite time resolution of the experimental apparatus, it is reasonable to assume that the strongly bound states rather than the weakly bound ones dominate the lifetime. We accordingly interpret the reported lifetime as the time that myosin is attached to actin in the strongly bound states. As a consequence, the reported duty ratio *r* ≃ 0.11 and force-free binding time in the strongly bound states *t*_sb_ ≃ 0.213 s provide an estimate of the time that the myosin molecule resides in the weakly bound states,

$$t_{\text{wb}}=t_{\text{sb}}\frac{1-r}{r}≃1.72\;s.$$ (30)

The rate constant, $\omega_{23}^{0}$ for phosphate release has been reported for human myosin-IC [82] as

$$\omega_{23}^{0}≃1.5\;s^{-1}.$$ (31)

Because of the opaque nomenclature of myosin-I isoforms, human myosin-IC is instead myosin Ie in the nomenclature of the Human Genome Organization [83]. This value is reasonable if the rate-limiting step is phosphate release and the order of magnitude is in agreement with the considerations for myosin Ic given in [19].

We next discuss the binding probability and the free-energy difference associated with the power stroke. Because there are to our knowledge no direct measurements for myosin Ic, we use values reported for myosin II. To estimate the probability *π*_2_ that the head binds in state (2), we refer to the cycle for rabbit skeletal muscle [2]. The reported numbers suggest a probability

$$\pi_{2}≃0.998,$$ (32)

which implies that myosin starts its cycle predominantly in state (2). Using the definition of this binding probability

$$\pi_{2}=\frac{\omega_{12}}{\omega_{12}+\omega_{15}},$$ (33)

we can relate the binding rates to each other as

$$\omega_{12}=\frac{\pi_{2}\omega_{15}}{1-\pi_{2}}.$$ (34)

Because both transition rates depend linearly on the actin concentration (Eqs 23 and 24), the actin concentration cancels and

$${\hat{\omega }}_{12}=\frac{\pi_{2}{\hat{\omega }}_{15}}{1-\pi_{2}}.$$ (35)

Several studies suggest that a large free-energy difference is associated with the main power stroke [2, 28, 32–34, 38]. Using the value Δ*G*_23_ ≃ −15 *k*_B_*T* inferred from fitting a model of the myosin II cycle to experimental data acquired with frog muscle [28], we obtain the ratio

$$\frac{\omega_{23}^{0}}{{\hat{\omega }}_{32}^{0}\left[P_{i}\right]}=\exp\left(-\Delta G_{23}/k_{B}T\right),$$ (36)

which provides the phosphate-binding rate constant

$${\hat{\omega }}_{32}^{0}=\frac{\omega_{23}^{0}}{\left[P_{i}\right]}\exp\left(\Delta G_{23}/k_{B}T\right).$$ (37)

Assuming a phosphate concentration of [P_i_] = 1 mM in frog muscle and using Eq 31, we determine that

$${\hat{\omega }}_{32}^{0}≃4.5\cdot 10^{-10}\;s^{-1}\mu M^{-1}.$$ (38)

A complementary approach is to use the values for the rabbit muscle cycle [2]; we then obtain ${\hat{\omega }}_{32}^{0}≃4.6\cdot 10^{-9}\;s^{-1}\mu M^{-1}$, a value one order of magnitude larger. Because both rate constants are very small compared to the other transition rates of our description, they make no significant difference in our results.

In a one-cycle description, the effects of changing the ATP and ADP concentrations are tightly coupled and determined by the magnitudes of the rate constants. If we assume a very fast and irreversible unbinding from the ATP state, *ω*_51_ ≫ 1 and *ω*_15_ = 0, and an irreversible P_i_ release, *ω*_32_ = 0, the average time in the strongly bound states reads

$$t_{\text{sb}}=\frac{\omega_{34}+\omega_{43}+\omega_{45}}{\omega_{34}\omega_{45}}.$$ (39)

In the force-free case the ADP and ATP binding rates are given in Eqs 21 and 22. Using the equilibrium binding constant *K*_ADP_ to estimate the rate constant for ADP binding as

$${\hat{\omega }}_{43}^{0}=\frac{\omega_{34}^{0}}{K_{\text{ADP}}},$$ (40)

we rewrite Eq 39 as

$$t_{\text{sb}}=\frac{\omega_{34}^{0}\left(1+\left[\text{ADP}\right]/K_{\text{ADP}}\right)+{\hat{\omega }}_{45}^{0}\left[\text{ATP}\right]}{\omega_{34}^{0}{\hat{\omega }}_{45}^{0}\left[\text{ATP}\right]}.$$ (41)

The two rate constants and the equilibrium constant have been determined experimentally as $\omega_{34}^{0}≃3.9\;s^{-1}$, ${\hat{\omega }}_{45}^{0}≃0.26\;s^{-1}\mu M^{-1}$ and *K*_ADP_ ≃ 0.22 *μ*M [20]. Because of the low ATP-binding rate constant and the small equilibrium constant *K*_ADP_ for ADP release, the lifetime of the strongly bound states is very sensitive to elevated ADP concentrations. Because the experimental findings differ, we resolve this problem in our one-cycle description by using the higher value of *K*_ADP_ ≃ 1.8 *μ*M for the equilibrium constant for ADP release [22, 23]. Although another possibility would be to introduce a multi-cycle description, that strategy increases complexity and the number of unknown parameters.

To satisfy thermodynamic consistency, we express the transition rate for ATP release in terms of all the other transition rates as given by the balance condition of Eq 29,

$$\omega_{54}=\frac{\omega_{12}\omega_{23}\omega_{34}\omega_{45}\omega_{51}}{\omega_{21}\omega_{32}\omega_{43}\omega_{15}}\exp\left(-\left(\Delta \mu -\left(\Delta x_{1}+\Delta x_{2}\right)F\right)/k_{B}T\right).$$ (42)

This transition rate is dependent on force in the same way as *ω*_51_ but independent of the nucleotide concentrations, as can be concluded by applying eqs (12)–(16), (20)–(22) and (27), resulting in

$$\omega_{54}=\frac{\omega_{12}\omega_{23}^{0}\omega_{34}^{0}{\hat{\omega }}_{45}^{0}g\left(F\right)\omega_{51}^{0}}{\omega_{21}{\hat{\omega }}_{32}^{0}{\hat{\omega }}_{43}^{0}\omega_{15}K_{\text{eq}}}\equiv \omega_{54}^{0}g\left(F\right).$$ (43)

We are left with seven unknown parameter values: the unbinding rates *ω*_51_ and *ω*_21_, the binding rate *ω*_15_, the force distribution factors *δ*_1_ and *δ*_2_, the characteristic length *ξ*, and the offset rate *ω*_off_. To estimate these values, we use analytic expressions of the average time spent in the weakly bound states and the effective unbinding rate from the strongly bound states and fit these functions to the experimental data. We derive these analytic expressions in the following section.

### Quantities of the cross-bridge cycle

The probability *P*_*i*_ of finding myosin in one of the states of the cycle is given by the solution to the steady-state master equation

$$0=\left(\begin{array}{ccccc} -\omega_{12}-\omega_{15} & \omega_{21} & 0 & 0 & \omega_{51}\\ \omega_{12} & -\omega_{21}-\omega_{23} & \omega_{32} & 0 & 0\\ 0 & \omega_{23} & -\omega_{32}-\omega_{34} & \omega_{43} & 0\\ 0 & 0 & \omega_{34} & -\omega_{43}-\omega_{45} & \omega_{54}\\ \omega_{15} & 0 & 0 & \omega_{45} & -\omega_{54}-\omega_{51} \end{array}\right)\left(\begin{array}{c} P_{1}\\ P_{2}\\ P_{3}\\ P_{4}\\ P_{5} \end{array}\right),$$ (44)

and read

$$P_{1}\equiv (\omega_{23}\omega_{34}\omega_{45}\omega_{51}+\omega_{21}\left(\omega_{34}\omega_{45}\omega_{51}+\omega_{32}\left(\omega_{43}+\omega_{45}\right)\omega_{51}+\omega_{32}\omega_{43}\omega_{54}\right)/N,$$ (45)

$$P_{2}\equiv \left(\omega_{12}\left(\omega_{34}\omega_{45}+\omega_{32}\left(\omega_{43}+\omega_{45}\right)\right)\omega_{51}+\left(\omega_{12}+\omega_{15}\right)\omega_{32}\omega_{43}\omega_{54}\right)/N,$$ (46)

$$P_{3}\equiv \left(\omega_{15}\left(\omega_{21}+\omega_{23}\right)\omega_{43}\omega_{54}+\omega_{12}\omega_{23}\left(\omega_{45}\omega_{51}+\omega_{43}\left(\omega_{51}+\omega_{54}\right)\right)\right)/N,$$ (47)

$$P_{4}\equiv \left(\omega_{15}\left(\omega_{23}\omega_{34}+\omega_{21}\left(\omega_{32}+\omega_{34}\right)\right)\omega_{54}+\omega_{12}\omega_{23}\omega_{34}\left(\omega_{51}+\omega_{54}\right)\right)/N,$$ (48)

$$P_{5}\equiv \left(\omega_{12}\omega_{23}\omega_{34}\omega_{45}+\omega_{15}\left(\omega_{21}\omega_{32}\omega_{43}+\omega_{23}\omega_{34}\omega_{45}+\omega_{21}\left(\omega_{32}+\omega_{34}\right)\omega_{45}\right)\right)/N,$$ (49)

in which N is determined by the normalization ∑_*i*_ *P*_*i*_ = 1.

**Fig 9:**
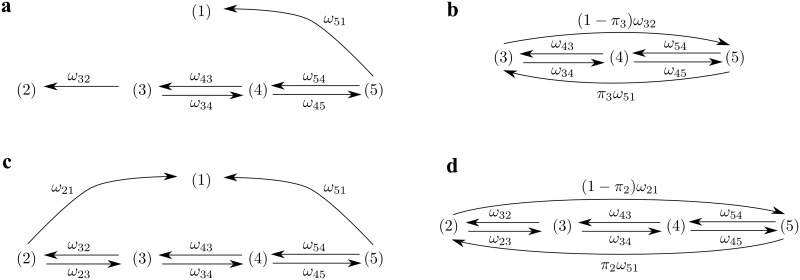
Diagram modified from Fig 1 to calculate the average time in different states. (a) To determine the time that myosin spends in the strongly bound states, we promote state (1) and (2) to absorbing states by eliminating the transitions from these states. (b) A closed diagram is obtained from (a) by redirecting the arrows into the absorbing states (1) and (2) back to the starting states (3) and (5) weighted with the starting probabilities *π*_3_ and (1 − *π*_3_), respectively. In the same manner, we construct the diagrams shown in (c) and (d) to determine the time that myosin is attached to actin.

To determine the effective unbinding rate from the strongly bound states, we calculate the average attachment time in the strongly bound states using a framework introduced by Hill [84, 85]. We promote the detached state (1) and weakly bound state (2) to absorbing states by setting the transition rates *ω*_15_, *ω*_23_, *ω*_12_, and *ω*_21_ to zero (Fig 9a). The associated effective unbinding rate then becomes the inverse of the average time to absorption for the appropriate initial condition. The basic idea is to use an ensemble average instead of a time average. The dynamics of the correct ensemble is described by a closed diagram in which the absorbing state is eliminated by redirecting the transitions into that state to the starting states weighted with the appropriate starting probabilities [86]. For example, the transition from state (3) to state (2) is redirected with the weight 1 − *π*_3_ to state (5) and with weight *π*_3_ to state (3). The latter transition, a self loop, cancels in a master equation and can therefore be disregarded. This procedure creates a closed diagram (Fig 9b) for which the steady-state probability *p*_*i*_ of being in state (*i*) is determined from the master equation

$$0=\left(\begin{array}{ccc} -\left(1-\pi_{3}\right)\omega_{32}-\omega_{34} & \omega_{43} & \pi_{3}\omega_{51}\\ \omega_{34} & -\omega_{43}-\omega_{45} & \omega_{54}\\ \left(1-\pi_{3}\right)\omega_{32} & \omega_{45} & -\omega_{54}-\pi_{3}\omega_{51} \end{array}\right)\left(\begin{array}{c} p_{3}\\ p_{4}\\ p_{5} \end{array}\right),$$ (50)

together with the normalization condition ∑*p*_*i*_ = 1. This probability distribution provides the average state occupancy before absorption. The average rate of arrivals at either of the absorbing states is therefore given by the probability current, which is identical to the effective unbinding rate from the strongly bound states

$$t_{\text{sb}}^{-1}\equiv \omega_{32}p_{3}+\omega_{51}p_{5}.$$ (51)

The probability of starting in state (3) and not in state (5) is given by the relative probability current into state (3) of the complete cycle as

$$\pi_{3}\equiv \frac{\omega_{23}P_{2}}{\omega_{23}P_{2}+\omega_{15}P_{1}},$$ (52)

in which *P*_1_ and *P*_2_ are given in Eqs 45 and 46, respectively. For the sake of completeness we give the rather cumbersome expression for the effective unbinding rate from the strongly bound states,

$$\begin{array}{l} t_{\text{sb}}^{-1}=(((\omega_{\text{15}}\omega_{\text{21}}\;\text{+}\;\omega_{12}\omega_{23})\omega_{32}\omega_{43}\;+\;(\omega_{12}\omega_{23}(\omega_{32}\;+\;\omega_{34})\;+\;\omega_{15}(\omega_{21}\omega_{32}\;+\;(\omega_{21}\\ \;+\;\omega_{23})\omega_{34}))\omega_{45})\omega_{51}\;+\;(\omega_{15}\omega_{21}\;+\;(\omega_{12}\;+\;\omega_{15})\omega_{23})\omega_{32}\omega_{43}\omega_{54})/H, \end{array}$$ (53)

in which

$$\begin{array}{l} H\equiv \omega_{15}(\omega_{21}(\omega_{32}(\omega_{43}\;+\omega_{45})+\omega_{34}\omega_{45})+\omega_{23}\omega_{34}\omega_{45}+(\omega_{23}(\omega_{34}+\omega_{43})+\omega_{21}(\omega_{32}\\ \;+\omega_{34}+\omega_{43}))\omega_{54})+\omega_{12}\omega_{23}((\omega_{43}\;+\omega_{45})\omega_{51}+\omega_{43}\omega_{54}+\omega_{34}(\omega_{45}+\omega_{51}+\omega_{54})). \end{array}$$ (54)

The time that myosin spends in the weakly bound states can be obtained from Eq 3 as

$$t_{\text{wb}}=t_{\text{sb}}\left(\frac{1}{\sum_{i=3}^{5}P_{i}}-1\right),$$ (55)

in which *P*_*i*_ are the steady-state probabilities given in Eqs 47–49.

To determine the time during which myosin is attached to the filament, we promote state (1) to an absorbing state. The corresponding closed diagram is obtained by redirecting the transition from state (2) to state (1) with weight 1 − *π*_2_ to state (5) and the transition from state (5) to state (1) with weight *π*_2_ to state (2). The steady-state probability *s*_*i*_ of being in state (*i*) for this closed diagram is the solution of the master equation

$$0=\left(\begin{array}{cccc} -\omega_{23}-\left(1-\pi_{2}\right)\omega_{21} & \omega_{32} & 0 & \pi_{2}\omega_{51}\\ \omega_{23} & -\omega_{32}-\omega_{34} & \omega_{43} & 0\\ 0 & \omega_{34} & -\omega_{43}-\omega_{45} & \omega_{54}\\ \left(1-\pi_{2}\right)\omega_{21} & 0 & \omega_{45} & -\omega_{54}-\pi_{2}\omega_{51} \end{array}\right)\left(\begin{array}{c} s_{2}\\ s_{3}\\ s_{4}\\ s_{5} \end{array}\right),$$ (56)

together with the normalization condition ∑*s*_*i*_ = 1. This probability distribution gives the probability current into state (1), which is identical to the unbinding rate from the actin filament

$$k_{\text{off}}\equiv \omega_{21}s_{2}+\omega_{51}s_{5}.$$ (57)

Again, for completeness, we give the unwieldy expression for the unbinding rate from the filament,

$$k_{\text{off}}=\frac{\left(\omega_{23}\omega_{34}\omega_{45}+\omega_{21}\left(\omega_{34}\omega_{45}+\omega_{32}\left(\omega_{43}+\omega_{45}\right)\right)\right)\omega_{51}+\omega_{21}\omega_{32}\omega_{43}\omega_{54}}{A+B+C},$$ (58)

in which

$$A\equiv \pi_{2}\left(\omega_{34}\omega_{45}+\omega_{32}\left(\omega_{43}+\omega_{45}\right)\right)\omega_{51}+\omega_{32}\omega_{43}\omega_{54},$$ (59)

$$B\equiv \left(1-\pi_{2}\right)\omega_{21}\left(\omega_{34}\omega_{45}+\left(\omega_{34}+\omega_{43}\right)\omega_{54}+\omega_{32}\left(\omega_{43}+\omega_{45}+\omega_{54}\right)\right),$$ (60)

$$C\equiv \omega_{23}\left(\pi_{2}\left(\omega_{43}+\omega_{45}\right)\omega_{51}+\omega_{43}\omega_{54}+\omega_{34}\left(\omega_{45}+\pi_{2}\omega_{51}+\omega_{54}\right)\right),$$ (61)

$$\pi_{2}\equiv \omega_{12}/\left(\omega_{12}+\omega_{15}\right).$$ (62)

Note that *π*_2_ is independent of the time *t*_off_ during which myosin is detached from the filament, and therefore independent of the actin concentration. As a consequence the unbinding rate *k*_off_ is also independent of the actin concentration.

#### Fit to the experimental data

The experimental data presented in this paper have been published [20] and are used with the kind permission of M. J. Greenberg. In these experiments the attachment time of a single myosin Ic head to actin has been measured under different loading forces and two different nucleotide concentrations: [ATP] ≃ 5 mM and [ATP] ≃ 1 mM with [ADP] ≃ 50 *μ*M. In both cases we assume a small contamination by phosphate, [*P*_*i*_] = 0.1 *μ*M, and in addition for the first case [ADP] = 0.1 *μ*M. Although it is very likely that the contamination is even lower, using a smaller value did not significantly change the numbers that we report here. The available data consist of measurements of the duration of actomyosin attachment and the associated force applied by the isometric optical clamp (Fig. 2A in ref. [20]). To obtain the unbinding rate as a function of force, we binned the attachment durations according to their forces in 1 pN bins and calculated for each bin the inverse of the average attachment time (Fig 2a). Because of the limited temporal resolution in the experiments, the recorded attachment events correspond to binding into the strongly bound states. We therefore consider the experimental unbinding rate as the effective unbinding rate from the strongly bound states.

To estimate the confidence intervals, we assumed that the measured binding times follow an exponential distribution for which the upper confidence interval boundary is given by $\frac{t_{\text{sb}}^{-1}\chi_{1-\alpha /2,2n}^{2}}{2n}$ and the lower boundary by $\frac{t_{\text{sb}}^{-1}\chi_{\alpha /2,2n}^{2}}{2n}$ [87]. Here $t_{\text{sb}}^{-1}$ is the average value, *n* the number of samples, and $\chi_{\mu ,\nu }^{2}$ the 100 ⋅ *μ* percentile of the *χ*^2^ distribution with *μ* degrees of freedom. To obtain the 95% confidence intervals we set *α* = 0.05.

We applied a least-square minimization weighted with the confidence intervals and simultaneously fit the data sets for the two different nucleotide concentrations (Fig 2). The numerical values obtained in this way are given in Table 1. The residence time in the strongly bound states does not depend on how long the molecule resides in state (1), which is governed by *ω*_15_. We therefore set *ω*_15_ to an arbitrary value of 1 s^−1^ for our fitting procedure. After determining all the other parameter values, we fit the time that myosin spends in the weakly bound states *t*_wb_ from Eq 55 to the experimental value from Eq 30 to determine *ω*_15_.

### Ensemble of cooperating myosin molecules

To describe the ensemble of myosins as a Markov chain, we introduce a state space (Fig 6a) associated with the number of bound myosin heads [47]. Assuming that the heads bind and unbind independently of one another, transitions between these states can be expressed in terms of the individual binding and unbinding rates *k*_on_ and *k*_off_. A transition from state (*n*), in which *n* myosins are bound, to state (*n* + 1) is associated with the binding rate

$$k_{\text{on}}^{n}\equiv \left(N-n\right)k_{\text{on}}.$$ (63)

The reverse transition is described by the unbinding rate

$$k_{\text{off}}^{n}\equiv nk_{\text{off}}.$$ (64)

We next incorporate an external force *F* into the description. As a first approximation, we assume that the myosin heads share this load equally, resulting in an effective force *F*/*n* exerted on each of the bound myosin heads and in a modified transition rate

$$k_{\text{off}}^{n}\equiv nk_{\text{off}}\left(F/n\right).$$ (65)

With this specific choice of the transition rates we determine the probability *S*_*n*_ of being in state (*n*) from a master equation. The solution for such a finite linear Markov chain is a standard result in stochastic dynamics and can be obtained recursively or by standard methods [47, 81]. The probability of being in state (*n*) is

$$S_{n}=S_{0}\prod_{i=0}^{n-1}\frac{k_{\text{on}}^{i}}{k_{\text{off}}^{i+1}},$$ (66)

in which *S*_0_ is determined from the normalization ∑*S*_*i*_ = 1 as

$$S_{0}={\left[1+\sum_{n=0}^{N-1}\prod_{i=0}^{n}\frac{k_{\text{on}}^{i}}{k_{\text{off}}^{i+1}}\right]}^{-1}.$$ (67)

From this probability distribution we obtain the average number of bound myosin heads as

$$\langle n\rangle =\sum_{n=0}^{N}nS_{n}.$$ (68)

### Monte Carlo simulations of an ensemble of elastically coupled myosins

We describe the dynamics of a myosin head with the five state chemomechanical cycle that we developed in this study. Each myosin head is coupled with a spring to a rigid common structure. The extension of this spring determines the force that is exerted on the myosin molecule and thus all transition rates of its cycle. We assume that a myosin head binds without tension to the actin filament and when it proceeds through its cycle the power-stroke transitions stretches the spring. At each time step we determine the extensions of all myosin springs from Newton’s law of force balance and adjust all transition rates accordingly. For the Monte Carlo simulations we use a Gillespie algorithm which is a standard method to simulate multi-component stochastic reactions and used for elastically coupled motor molecules [35, 78, 79, 88]. After disregarding the transient behavior of our simulations, we determine the average number of myosins bound to actin from a time average. We consider all myosin molecules in state (2) to (5) bound to actin.
